# *Superficieibacter electus* gen. nov., sp. nov., an Extended-Spectrum β-Lactamase Possessing Member of the Enterobacteriaceae Family, Isolated From Intensive Care Unit Surfaces

**DOI:** 10.3389/fmicb.2018.01629

**Published:** 2018-07-20

**Authors:** Robert F. Potter, Alaric W. D'Souza, Meghan A. Wallace, Angela Shupe, Sanket Patel, Danish Gul, Jennie H. Kwon, Wandy Beatty, Saadia Andleeb, Carey-Ann D. Burnham, Gautam Dantas

**Affiliations:** ^1^The Edison Family Center for Genome Sciences and Systems Biology, Washington University in St. Louis School of Medicine, St. Louis, MO, United States; ^2^Department of Pathology and Immunology, Washington University in St. Louis School of Medicine, St. Louis, MO, United States; ^3^Atta ur Rahman School of Applied Biosciences, National University of Sciences and Technology, Islamabad, Pakistan; ^4^Division of Infectious Diseases, Washington University School of Medicine, St. Louis, MO, United States; ^5^Department of Molecular Microbiology, Washington University in St. Louis School of Medicine, St. Louis, MO, United States; ^6^Department of Pediatrics, St. Louis Children's Hospital, St. Louis, MO, United States; ^7^Department of Biomedical Engineering, Washington University in St. Louis, St. Louis, MO, United States

**Keywords:** ESBL harboring bacteria, Enterobacteriaceae taxonomy, Hospital surface surveillance, phylogenomics, antibiotic resistance genes

## Abstract

Two Gram-negative bacilli strains, designated BP-1(T) and BP-2, were recovered from two different Intensive Care Unit surfaces during a longitudinal survey in Pakistan. Both strains were unidentified using the bioMerieux VITEK MS IVD v2.3.3 and Bruker BioTyper MALDI-TOF mass spectrometry platforms. To more precisely determine the taxonomic identity of BP-1(T) and BP-2, we employed a biochemical and phylogenomic approach. The 16S rRNA gene sequence of strain BP-1(T) had the highest identity to *Citrobacter farmeri* CDC 2991-81(T) (98.63%) *Citrobacter amalonaticus* CECT 863(T) (98.56%), *Citrobacter sedlakii* NBRC 105722(T) (97.74%) and *Citrobacter rodentium* NBRC 105723(T) (97.74%). The biochemical utilization scheme of BP-1(T) using the Analytic Profile Index for Enterobacteriaceae (API20E) indicated its enzymatic functions are unique within the Enterobacteriaceae but most closely resemble *Kluyvera* spp., *Enterobacter cloacae* and *Citrobacter koseri/farmeri*. Phylogenomic analysis of the shared genes between BP-1(T), BP-2 and type strains from *Kluyvera, Citrobacter, Escherichia, Salmonella, Kosakonia, Siccibacter* and *Shigella* indicate that BP-1(T) and BP-2 isolates form a distinct branch from these genera. Average Nucleotide Identity analysis indicates that BP-1(T) and BP-2 are the same species. The biochemical and phylogenomic analysis indicate strains BP-1(T) and BP-2 represent a novel species from a new genus within the Enterobacteriaceae family, for which the name *Superficieibacter electus* gen. nov., sp. nov., is proposed. The type strain is BP-1(T) (= ATCC BAA-2937, = NBRC 113412).

## Introduction

The hospital built environment is a key source of nosocomial pathogens, which can cause high mortality infections in vulnerable patient populations (Oberauner et al., 2013). The danger from these infections is exacerbated by the higher levels of antibiotic resistance harbored by nosocomial pathogens compared to their community-associated relatives (Horcajada et al., 2013). As many pathogens can persist on surfaces for extended periods of time, microbiological and molecular surveillance of high-contact surfaces in health-care settings is an important feature of effective infection control and prevention strategies (Russotto et al., 2015). The isolates reported herein were recovered as part of such a surveillance program of intensive care unit (ICU) surfaces at a tertiary care hospital in Pakistan in 2016.

The Enterobacteriaceae is a diverse family of Gammaproteobacteria that includes many common human pathogens (Paradis et al., 2005; Potter et al., 2016). Enterobacteriaceae taxonomy is complicated, as demonstrated by the use of multilocus sequence analysis to reclassify several *Enterobacter* species into the *Lelliottia, Pluralibacter* and *Kosakonia* genera (Brady et al., 2013). Next-generation sequencing has significantly improved our understanding of Enterobacteriaceae taxonomy by identifying similarities between species of different genera (e.g., *Salmonella subterranean* and *Escherichia hermannii* into *Atlantibacter subterranea* and *Atlantibacter hermannii*, respectively) or resolving closely related species within the same genus (e.g., *Klebsiella pneumoniae* and *Klebsiella variicola*) (Holt et al., 2015; Hata et al., 2016). Many Enterobacteriaceae strains are medically relevant not only as etiological agents of disease but also as reservoirs for transferable antibiotic resistance genes (Sirot et al., 1988). Thus, documenting the appearance of novel Enterobacteriaceae species in hospital settings is an important component in surveilling and preventing emerging infectious diseases (Iredell et al., 2016).

## Materials and methods

### Bacterial isolation

We isolated strain BP-1(T) (bedside rail) and BP-2 (nurse call button) from an intensive care room at a tertiary care hospital in Pakistan with the Eswab collection device and transport system (Becton Dickenson & Company, Franklin Lakes, NJ) in June 2016. Surfaces were swabbed in triplicate during an ongoing yearlong longitudinal sampling study. Swabs were sent to the US at room temperature and arrived in the US within ~3 days of sampling. Samples from Pakistan were received by the Burnham lab under approval of the Washington University IBC Protocol number 6,572. Swabs were cultured within 24 h of arrival in the US laboratory site. The isolates were recovered on MacConkey agar with 5 μg/ml cefotaxime (Hardy Diagnostics, Santa Maria, CA) and re-streaked on blood agar using the four-quadrant method to recover distinct single colonies. The isolates were unidentified by the MALDI-TOF VITEK MS IVD v2.3.3 (bioMerieux, Durham, NC) and Bruker BioTyper (Bruker Daltonics, Billerica, MA) mass spectrometry systems. For subsequent genomic analysis, frozen stocks of each isolate were made by creating a dense suspension of the isolate in Tryptic Soy Broth (Sigma Aldrich, St. Louis, MO) with 15% Glycerol (Hardy Diagnostics, Santa Maria, CA).

### Biochemical tests and growth assays

Glycerol stocks of strain BP-1(T) and BP-2 were streaked separately onto 5% sheep′s blood agar (Hardy Diagnostics, Santa Maria, CA), HardyCHROM™ ESBL Agar (Hardy Diagnostics, Santa Maria, CA), Hektoen Enteric Agar (Remel, Lenexa, KS) and MacConkey agar (Hardy Diagnostics, Santa Maria, CA) plates using four quadrant streaking. Plates were incubated for 35°C in air and then imaged. Additional streaks of blood agar plates were incubated at either 4, 25, 35, or 42°C. Blood agar plates were also stored in an anaerobic bag (BD, Franklin Lakes, NJ) at 35°C. Phenotypic assays to assess indole production, catalase, urease and motility were performed. The isolates were also evaluated on lysine iron agar (LIA) (Remel, Lenexa, KS), triple sugar iron (TSI) agar (Remel, Lenexa, KS), Bile esculin agar (Remel, Lenexa, KS) and with an oxidation/fermentation glucose test (OF glucose) (Remel, Lenexa, KS), all performed according to manufacturer′s recommendations. Each isolate was also evaluated using the Analytical Profile Index (API®) 20-E Enterobacteriaceae identification kit (bioMerieux, Durham, NC). API values of related taxa were obtained from for *Kluyvera, Enterobacter cloacae* and *Citrobacter koseri* (referred to as *Citrobacter amalonaticus* biogroup 1 by Farmer et al., 1981, 1985). Similar to how this information was used for *Escherichia albertii* delineation, characters are scored as “+” if > = 85% of the strains are positive, “−” if > = 85% of the strains are negative, “v+” if 50–85% of strains are positive and “v–” if 50-85% of strains are negative (Huys et al., 2003).

### Transmission electron microscopy

Glycerol stocks of strain BP-1(T) and BP-2 were plated onto MacConkey agar with ceftriaxone (5 μg/ml) overnight. A single colony of each isolate was separately inoculated into 5 ml of LB broth and grown until log phase at 37°C with orbital shaking at 220 rpm.

For analysis of whole bacteria by negative staining, the isolates absorbed onto formvar/carbon-coated copper grids for 2 min. Grids were washed in dH_2_O and stained with 1% aqueous uranyl acetate (Ted Pella Inc., Redding CA) for 1 min. Excess liquid was gently wicked off and grids were air dried. Bacteria viewed on a JEOL 1200 EX transmission electron microscope (JEOL USA Inc., Peabody, MA) equipped with an AMT 8-megapixel digital camera (Advanced Microscopy Techniques, Woburn, MA).

For ultrastructural analysis of cross-sections of bacteria, the isolates were fixed in 2% paraformaldehyde/2.5% glutaraldehyde in 100 mM cacodylate buffer, pH 7.2 for 1 h at room temperature. Samples were washed in cacodylate buffer and postfixed in 1% osmium tetroxide (Polysciences Inc.) for 1 h. Samples were then rinsed extensively in dH_2_0 prior to en bloc staining with 1% aqueous uranyl acetate (Ted Pella Inc., Redding, CA) for 1 h. Following several rinses in dH_2_0, samples were dehydrated in a graded series of ethanol and embedded in Eponate 12 resin (Ted Pella Inc.). Sections of 95 nm were cut with a Leica Ultracut UCT ultramicrotome (Leica Microsystems Inc., Bannockburn, IL), stained with uranyl acetate and lead citrate and viewed on the transmission electron microscope.

### Illumina whole genome sequencing

Genomic DNA was extracted using the bacteremia kit (Qiagen, Germantown, MD), from a suspension of ~10 colonies of strain BP-1(T) and BP-2 after overnight growth on blood agar (Hardy Diagnostics, Santa Maria, CA). 0.5 ng of DNA was used as input for constructing Nextera Illumina sequencing libraries (Illumina, San Diego, CA) (Baym et al., 2015). Sample libraries were sequenced on an Illumina NextSeq 550 to obtain ~2.5 million 2 × 150 bp reads. Raw reads had Illumina adapters removed with Trimmomatic and were decontaminated with Deconseq using the commands: “java -Xms1024m -Xmx1024m -jar $TRIMMOMATIC_HOME/trimmomatic-0.36.jar PE -phred33 -trimlog < trimlog.txt> < input forward reads> < input reverse reads> < output paired forward reads> < output unpaired forward reads> < output paired reverse reads> < output unpaired reverse reads> ILLUMINACLIP:/opt/apps/trimmomatic/0.36/adapters/NexteraPE-PE.fa:2:30:10:1:true” and “deconseq.pl -f < paired input reads> -o < paired output reads> -dbs hsref,” respectively (Schmieder and Edwards, 2011; Bolger et al., 2014). Paired forward and reverse reads were used as input for de-novo assembly with SPAdes with the command: “spades.py -k 21,33,55,77 –careful –pe2-1 < input forward reads> –pe2-2 < input reverse reads> -o < output directory>” (Bankevich et al., 2012). Assembly metrics were assessed by using QUAST on all contigs >500 bp in length using the webserver (http://quast.bioinf.spbau.ru/) (Gurevich et al., 2013). Draft whole genome sequences for strain BP-1(T) and BP-2 have been deposited with NCBI BioProject: PRJNA395420.

### 16S rRNA similarity

16S rRNA sequences from the BP-1(T) file were identified using Barrnap with the command: “barrnap –quiet < contigs.fasta>” (https://github.com/tseemann/barrnap) and manually retrieved. The sequence is on contig_69 from base pairs 22 to 1559. The complete 16S rRNA sequence was submitted to the EzBioCloud identify service on 08/31/17 (Kim et al., 2014; Yoon et al., 2017). The 16S rRNA sequences for the top 10 most similar taxa were retrieved and aligned using MUSCLE (Edgar, 2004) using the command “muscle -in < input_multifasta> -out < output_aligned_multifasta>.” The aligned 16S rRNA multifasta was made into a phylogenetic tree with 1,000 bootstraps using raxML with the command: “raxmlHPC -s < input_aligned_multifasta> -n output -m GTRGAMMA -p 100 -f a -N 1000 -x 12345.” The resulting newick tree was visualized in FigTree with bootstrap values depicted as node label. The aligned multifasta sequences were uploaded into Jalview v2.0 and colored by nucleotide (Waterhouse et al., 2009). The 16S rRNA sequence has been submitted to Genbank under accession number MG866003.

### Core genome phylogeny

Fifty-six genomes from type strains in species of *Escherichia, Shigella, Citrobacter, Salmonella, Kluyvera, Klebsiella, Kosakonia, Siccibacter* and *Pasteurella* (Table S1) were obtained from NCBI Genomes on 09/01/17. The protein coding sequences of these genomes and strains BP-1(T) and BP-2 were annotated using Prokka with the previously described command (Seemann, 2014). The core genome, representing genes shared by 100% of all isolates in the cohort was constructed and aligned using the PRANK module within roary with the following command: “roary -e –group_limit 700000 -p 8 -i < percent identity> ^*^.gff” (Page et al., 2015). Eighty percent of gene identity was used for core genome alignment of Enterobacteriaceae genomes plus the *P. multocida* outgroup and for core genome alignment of only the Enterobacteriaceae genomes.

Aligned multifasta files of the 48 genes shared by all Enterobacteriaceae genomes and *P. multocida* (Table S2) was constructed into a maximum likelihood phylogenetic tree using 1,000 bootstraps in RAxML with the following command: “raxmlHPC -s core_gene_alignment.aln -n output -m GTRGAMMA -o < *P. multocida* name>-p 100 -f a -N 1000 -x 12345” (Stamatakis, 2014). Trees were visualized in FigTree (http://tree.bio.ed.ac.uk/software/figtree/) with bootstrap values depicted as node labels.

Aligned multifasta files of the 750 genes shared by all Enterobacteriaceae genomes (Table S2) was constructed into a NeighborNet tree using the EqualAngle method in SplitsTree4 (v4.14.5) (Huson and Bryant, 2006).

### Average nucleotide identity

Whole genome sequences of strains BP-1(T) and BP-2 were compared against each other using the mummer method of average nucleotide identity via the jspecies webserver (http://jspecies.ribohost.com/jspeciesws/) on 09/18/17 (Richter et al., 2016). The contig file for strains BP-1(T), BP-2 and the set of Enterobacteriaceae genomes (Table S1) were compared using the mummer method of average nucleotide identity via the standalone pyANI tool (https://github.com/widdowquinn/pyani) with command “python3 average_nucleotide_identity.py -i < input_fasta_directory> -o < outout_directory> -m ANIm –nucmer_exe < mummer_path> -v –f.” The Hadamard matrix representing the multiplication of the percentage identity and alignment length was converted into a dendrogram using scipy.cluster.hierarchy.dendrogram. within python3 using “euclidian” metric and “weighted” method. The dendrogram was viewed in matplotlib.

### Phenotypic antibiotic susceptibility testing

Antimicrobial susceptibility testing was performed on the isolates using Kirby Bauer disk diffusion on Mueller Hinton Agar (Hardy Diagnostics, Santa Maria, CA) according to CLSI standards (CLSI, 2013). Results were interpreted using CLSI M100 criteria for Enterobacteriacace (CLSI, 2013).

### Antibiotic resistance gene identification and visualization

Antibiotic resistance genes (ARGs) from the draft whole genome assemblies of strains BP-1(T) and BP-2 were annotated by submission to the ResFinder webserver (https://cge.cbs.dtu.dk/services/ResFinder/) on 05/18/17 (Zankari et al., 2012). We annotated sequence divergent ARGs using Resfams with the command “annotate_functional_selections.py –Resfams_only -contigs < contig file> -o < output directory>” (Gibson et al., 2015). Putative resistant determinants were then mapped to observed phenotypic resistance in accordance with Resfams and ResFinder classification. Contig_4 in the BP-1(T) assembly was submitted to BLASTN against the nr/nt database on 01/22/18 (Camacho et al., 2009).

Strain BP-1(T) and BP-2 were annotated for protein coding sequences by Prokka using the command: “Prokka < input fasta> < output directory>” (Seemann, 2014). Contigs 54 and 72 from BP-1(T) had BLAST similarity compared against Contig 42 from BP-2 using default settings on WebAct (webact.org/WebACT/generate) (Abbott et al., 2007). BLAST similarity was viewed using EasyFig for sequences with e value 0.001 and length >100 bp (Sullivan et al., 2011).

## Results

### Colony appearance and ultrastructural analysis

**Figure 1:**
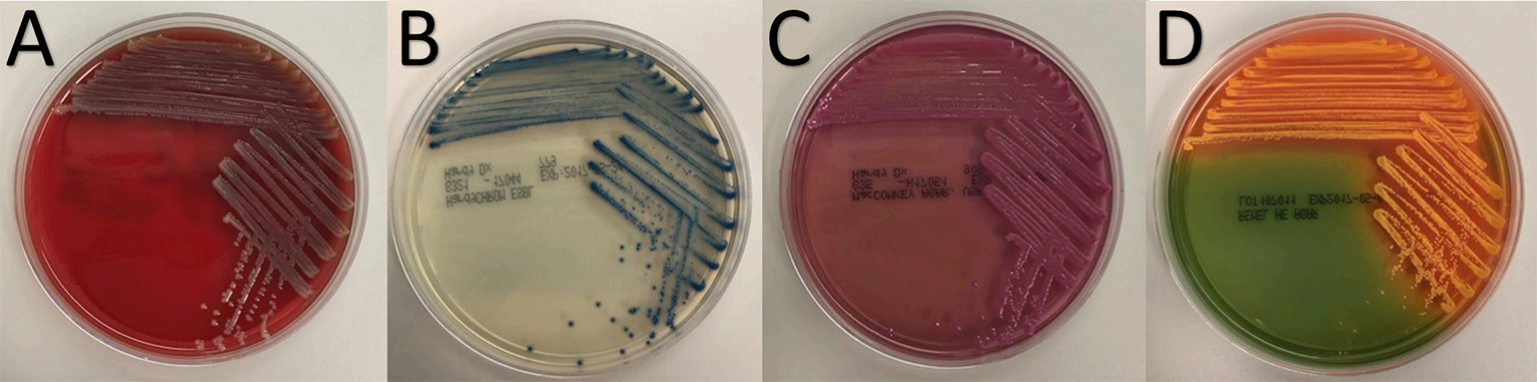
Colony morphology of strain BP-1(T) on **(A)** blood, **(B)** HardyCHROM™ ESBL, **(C)** MacConkey and **(D)** Hektoen Enteric agars after overnight growth at 35°C.

Both strains BP-1(T) and BP-2 appeared identical on agar plates. BP-1(T) formed circular, shiny, mucoid, gray colonies and were non-hemolytic on blood agar plates (Figure 1A). The colonies were small, blue and rigid on HardyCHROM™ ESBL Agar (Figure 1B). BP-1(T) colonies were bright pink on MacConkey agar (i.e., lactose fermenting) with an opaque zone surrounding the edge of the colony (Figure 1C). Coral colored colonies were produced on HE agar (Figure 1D).

**Figure 2:**
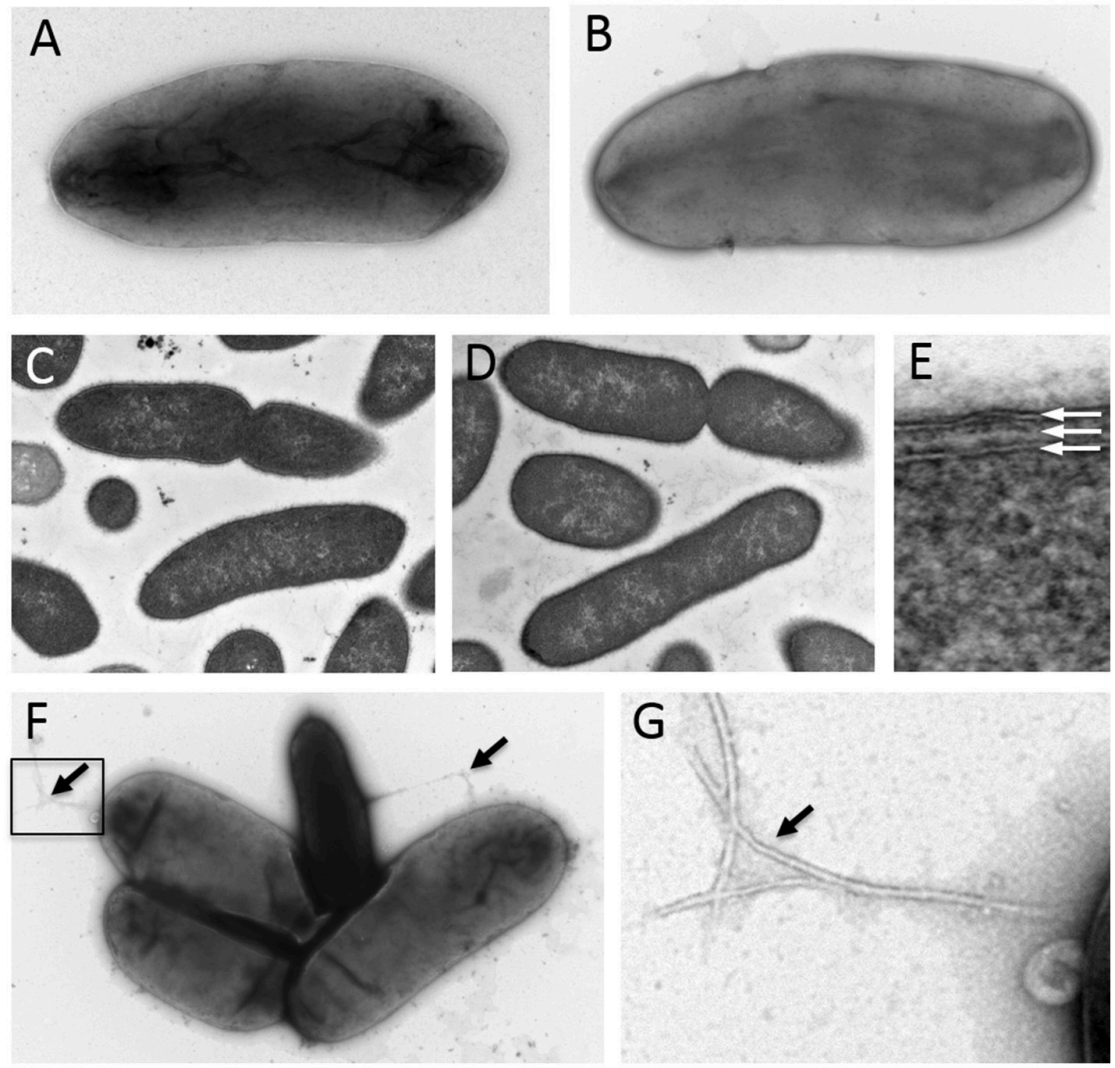
Negative staining of log-phase strains **(A)** BP-1(T) and **(B)** BP-2with 1% uranyl acetate show a bacilli shape structure ~2.7 μm in length. Cross sections of strains **(C)** BP-1(T) and **(D)** BP-2 from resin embedding identify rod shaped bacteria. The isolates have a double membrane structure characteristic with of Gram-negative bacteria **(E)**. The white arrows indicate the outer membrane, periplasm and cytoplasmic membrane. Negative staining showing several pilus-like structures (black arrows) protruding from strain BP-1(T) isolates (**F** and enlarged insert shown in **G**).

Analysis by negative staining and transmission electron microscopy revealed that BP-1(T) and BP-2 are rod shaped and ~2.7 μm in length (Figures 2A,B). Cross sections from resin embedding also show rod shaped bacteria (Figures 2C,D) with two electron dense lipid bilayers of the outer membrane and cytoplasmic membrane (Figure 2E). No evidence of flagella was observed, but pili-like structures were present between a group of isolates (Figures 2F,G).

### Biochemical and temperature studies

Strains BP-1(T) and BP-2 had identical results from phenotypic assays (Table 1). Aerobically, BP-1(T) showed no growth at 4°C, scant growth at 25 and 42°C, but robust growth at 35°C (Table 1). BP-1(T) and BP-2 both showed robust growth anaerobically at 35°C using an anaerobic bag (Table 1). The isolates were negative for motility, oxidase, indole, catalase and urease. Both strains BP-1(T) and BP-2 were positive for gas production in TSI and LIA reactions (Table 1). The strains grew on bile esculin agar and can ferment glucose (Table 1).

**Table 1:** Phenotypic evaluation of strains BP-1(T) and BP-2.

**Assay**	**BP-1(T)**	**BP-2**
**INTERPRETATION**		
Aerobic growth at 4°C	No growth	No Growth
Aerobic growth at 25°C	Scant growth	Scant growth
Aerobic growth at 42°C	Scant growth	Scant growth
Aerobic growth at 35°C	Robust growth	Robust growth
Anaerobic growth at 35°C	Robust growth	Robust growth
Oxidase	Negative	Negative
Indole production	Negative	Negative
Catalase	Positive	Positive
Urease	Negative	Negative
TSI reaction	A/A+ Gas (Acid/Acid)	A/A+ Gas (Acid/Acid)
LIA reaction	K/A+ gas (Alkaline/Acid)	K/A+ gas (Alkaline/Acid)
Bile esculin	+	+
OF glucose	Glucose fermenting	Glucose fermenting

As evaluated using the API 20E, BP-1(T) and BP-2 were positive for arabinose, amygdalin, melibiose, sacharose, rhamnose, sorbitol and mannitol fermentation, but negative for inositol fermentation (Table 2). BP-1(T) and BP-2 can utilize citrate and decarboxylate ornithine for metabolism in addition to utilizing disaccharides (melibiose) (Table 2). These biochemical test results were unable to assign a high confidence genus identification to strains BP-1(T) and BP-2. The analytic profile index (API) algorithm indicates that strains BP-1(T) and BP-2 most resembles *Kluyvera* spp. and to a lesser extent *E. cloacae* and *Citrobacter koseri/farmeri* (Table 3). Analysis of the biochemical utilization profile from these related taxa indicate that strains BP-1(T)/BP-2 and *Kluyvera cryocrescens* ATCC 33435 are both negative for arginine dihydrolase activity but *E. cloacae* and *C. koseri* are positive for it. Additionally, strains BP-1(T)/BP-2, E. cloacae and *C. koseri* can ferment D-sorbitol but *K. cryocrescens* ATCC 33435 cannot. Both BP-1(T) and BP-2 were tested as non-motile but *K. cryocrescens* ATCC 33435, *E. cloacae and C. koseri* are positive. Accordingly, BP-1(T)/BP-2 can be uniquely distinguished from its closest neighbors based on being negative for arginine hydrolase activity, positive for D-sorbitol fermentation and non-motile.

**Table 2:** Biochemical utilization of strains BP-1(T) and BP-2 with API20E.

**Reaction Tested**	**BP-1(T)**	**BP-2**	***K. cryocrescens* ATCC 33435**	***Enterobacter cloacae***	***Citrobacter koseri***
**INTERPRETATION**					
Beta-galactosidase	+	+	+	+	+
Arginine dihydrolase	–	–	–	+	+
Lysine decarboxylase	–	–	–	–	–
Ornithine decarboxylase	+	+	+	+	+
Citrate utilization	+	+	+	+	+
H2S production	–	–	–	–	–
Urease	–	–	–	v+	v+
Tryptophan deaminase	–	–	–^*^phenylalanine	–^*^phenylalanine	–^*^phenylalanine
Indole production	–	–	+	–	+
Acetoin production	–	–	–	+	–
Gelatinase	–	–	–	–	–
Glucose fermentation	+	+	+	+	+
Mannitol fermentation	+	+	+	+	+
Inositol fermentation	–	–	–	–	–
Sorbitol fermentation	+	+	–	+	+
Rhamnose fermentation	+	+	+	+	+
Sacharose fermentation	+	+	+	+	+
Melbiose fermentation	+	+	+	+	+
Amygdalin fermentation	+	+	N/A	N/A	N/A
Arabinose fermentation	+	+	+	+	+
Motility	–	–	+	+	+

* *Denotes that these isolates had phenylalanine deaminase activity measured*.

**Table 3:** API20E interpretation.

**Significant Taxa**	**Percent ID**
*Kluyvera* spp.	41.2
*Enterobacter cloacae*	30.7
*Citrobacter koseri/farmeri*	8.4
*Kluyvera intermedia*	8.1
*Serratia fonticola*	5.4

### Phylogenomic analysis

We used a phylogenomic approach to better delineate the taxonomic context of strains BP-1(T) and BP-2 within the Enterobacteriaceae. Submission of the 1,512-base pair 16S rRNA locus from strain BP-1(T) to the EzBioCloud taxonomic database indicated that strain BP-1(T) had the highest identity to *Citrobacter farmeri* CDC 2991-81(T), *C. amalonaticus* CECT 863(T), *C. sedlakii* NBRC 105722(T) and *C. rodentium* NBRC 105723(T) (Table 4). The maximum likelihood tree from alignment of these 16S rRNA sequences placed BP-1(T) in-between a group of *Citrobacter* species [*C. sedlakii* NBRC 105722(T), *C. rodentium* NBRC 105723(T), *C. farmeri* CDC 2991-81(T) and *C. amalonaticus* CECT 863(T)] and the other Enterobacteriaceae taxa including *Salmonella enterica* subsp. Arizonae ATCC 13314(T), *Kosakonia sacchari* SP1(T), *C. europaeus* 97/79(T), *Enterobacter hormaechei* susbp. steigerwaltii DSM 16691(T), an uncharacterized Enterobacter strain termed LGIT s RIT-PI-d and *Pluralibacter gergoviae* JCM 1234(T) (Figure S1). The greatest amount of variation in 16S rRNA identity occurred at the likely V3 region, around nucleotides 426–450 of the BP-1(T) sequence, using standard *E. coli* nomenclature (Chakravorty et al., 2007). The 6mer of GAGAAT in the BP-1(T) sequence from sites 426 to 435 is unique in comparison to the other sequences at this position. Although the GAG nucleotides from 426 to 428 are shared by *K. sacchari* SP1(T)/*P. gergoviae* JCM 1234(T) and the AT site at 434–435 is shared by *C. farmeri* CDC 2991-81^T^, the A residue at position 429 is discriminatory, as all other taxa contain a thymine at this site (Figure S2). As 98.7–98.65% is a proposed standard for species delineation via rRNA gene sequencing, we endeavored to gain further insight into the taxonomic identity of BP-1(T) and BP-2 based on their whole genome sequence characteristics.

**Table 4:** Top hits of strain BP-1(T) 16S rRNA sequence.

**Rank**	**Name**	**Strain**	**Authors**	**Accession**	**Pairwise similarity (%)**	**Mismatch/Total nt**	**Completeness (%)**
1	*Citrobacter farmeri*	CDC 2991-81	Brenner et al., 1993	AF025371	98.63013699	20/1460	100
2	*Citrobacter amalonaticus*	CECT 863	Young et al., 1971; Farmer, 1981	FR870441	98.56361149	21/1462	100
3	*Citrobacter sedlakii*	NBRC 105722	Brenner et al., 1993	BBNB01000023	97.74281806	33/1462	100
4	*Citrobacter rodentium*	NBRC 105723	Schauer et al., 1995	BBNA01000105	97.74281806	33/1462	100
5	*Salmonella enterica subsp. arizonae*	ATCC 13314	Tindall et al., 2005	AF008580	97.67282683	34/1461	100
6	*Citrobacter europaeus*	97/79	Ribeiro et al., 2017	LT615140	97.58190328	31/1282	87.56830601
7	*Enterobacter hormaechei subsp. steigerwaltii*	DSM 16691	Hoffmann et al., 2005	CP017179	97.5376197	36/1462	100
8	*Kosakonia sacchari*	SP1	Zhu et al., 2013; Gu et al., 2014	CP007215	97.53424658	36/1460	100

**Figure 3:**
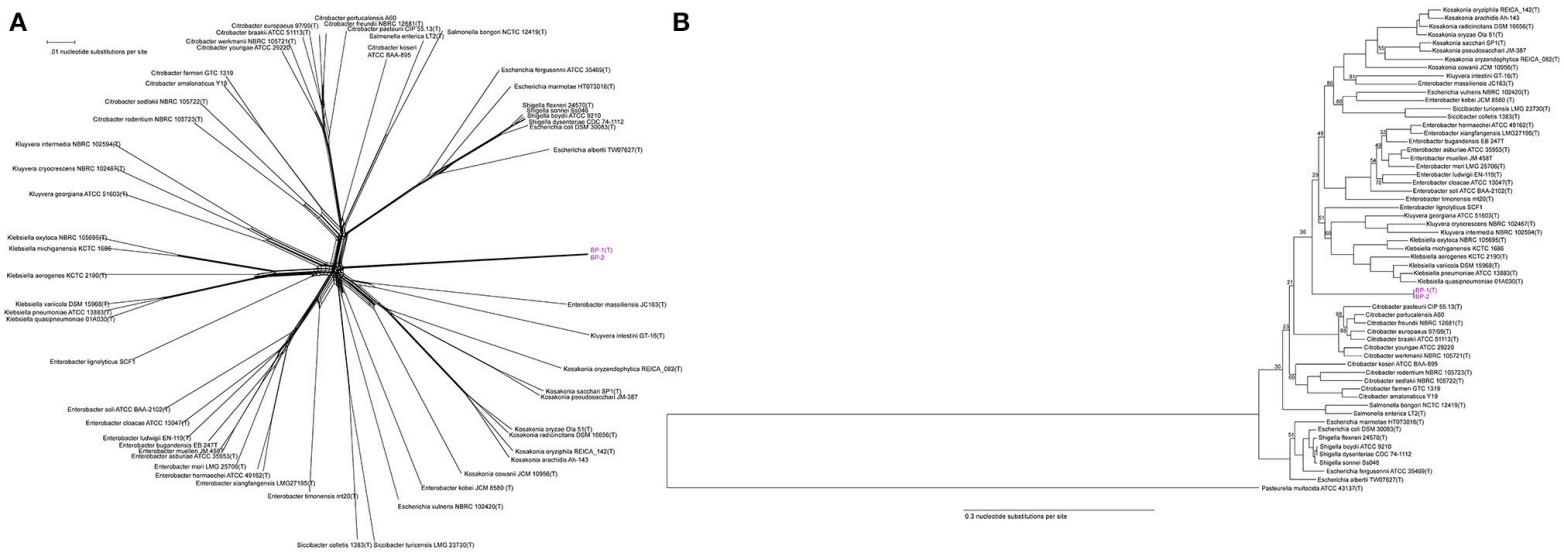
**(A)** Unrooted core genome NeighborNet tree from an alignment of the 750 genes shared by Enterobacteriaceae genomes with >80% nucleotide identity. Scale bar represents number of nucleotide substitutions. Strain BP-1(T) and BP-2 are a separate cluster from other Enterobacteriaceae genera. **(B)** Rooted Core genome phylogeny of Enterobacteriaceae genomes with an outgroup for comparison (*Pasteurella multocida*), based on 48 genes shared at >80% nucleotide identity by all genomes. Both strains BP-1(T) and BP-2 isolates clustered together in a clade separate from existing Enterobacteriaceae type strains. Bootstrap support values < 95 are depicted as branch labels. Scale bar represents number of nucleotide substitutions per site.

To determine the relative taxonomic identity of strains BP-1(T) and BP-2 beyond 16S rRNA similarity, we constructed an equal angle neighbor net tree produced from the aligned 750 core genes shared between type strains in *Enterobacter, Siccibacter, Escherichia, Enterobacter, Kosakonia, Kluyvera, Klebsiella, Shigella, Salmonella* and *Citrobacter* at >80% identity (Table S1). Analysis of the network indicates that strains BP-1(T) and BP-2 cluster together and distinctly from these major Enterobacteriaceae taxa (Figure 3A).

A maximum likelihood tree from the 48 genes shared between the same Enterobacteriaceae genomes and a *Pasteurella multocida* outgroup with >80% identity again placed strains BP-1(T) and BP-2 in a cluster separate from existing genera. The BP-1(T)/BP-2 branch separates the genomes analyzed into two major clades composed of *Escherichia* [excluding *Escherichia vulneris* NBRC 192420(T), which other groups have suggested is misclassified (Walk et al., 2009)], *Shigella, Citrobacter, Salmonella* in one branch and *Klebsiella, Kluyvera, Enterobacter, Siccibacter and Kosakonia* on the other branch (Figure 3B).

**Figure 4:**
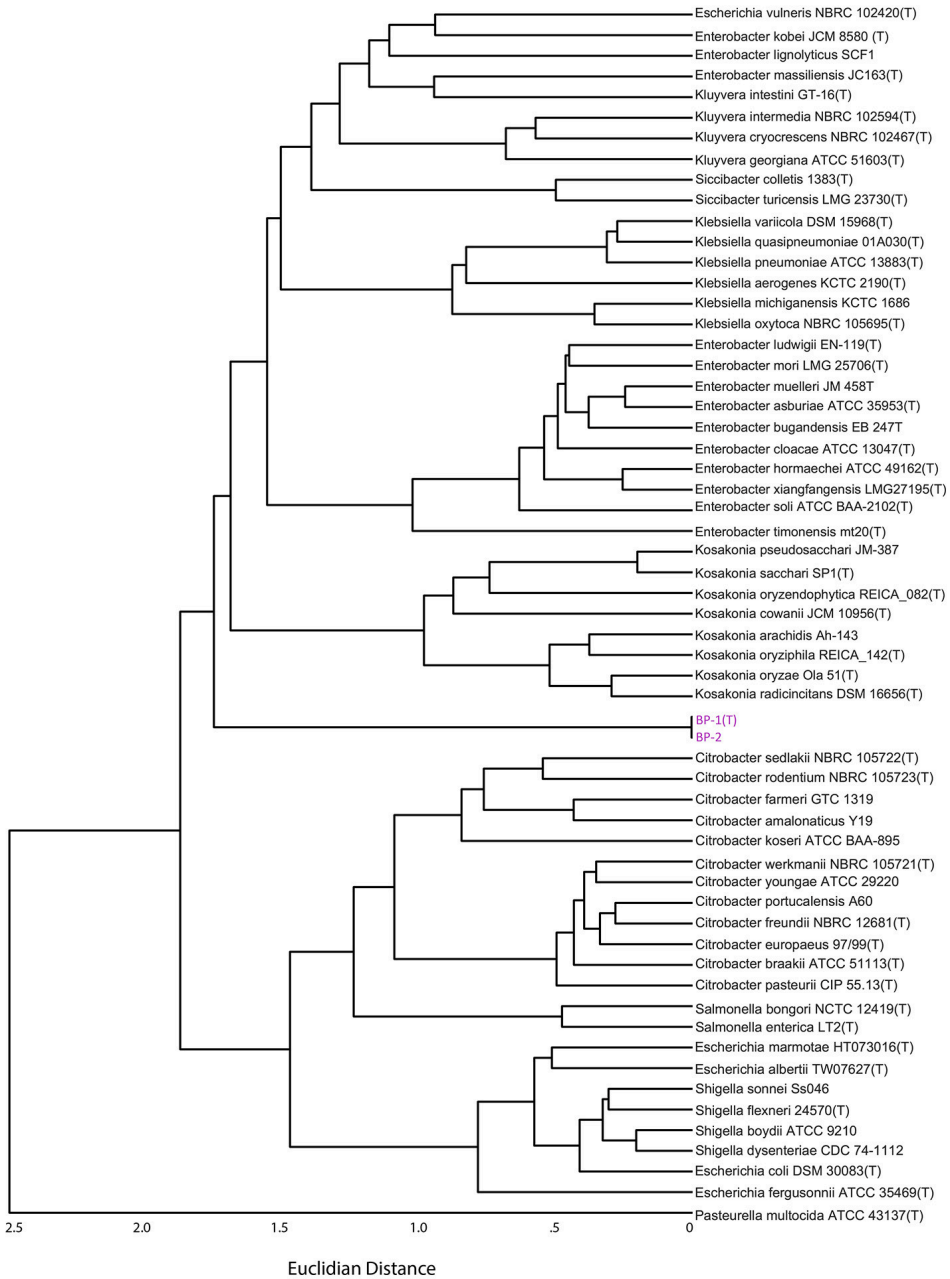
Dendrogram from the distance matrix representing the Hadamard matrix between average nucleotide identity and percent genome aligned. Strain BP-1 and BP-2 are split between two larger clades of Enterobacteriaceae bacteria, similar to Figure 3B.

Pairwise Average Nucleotide Identity (ANI) analysis using MUMer between strains BP-1(T) and BP-2 indicate that they are the same species, as the ANI value of 99.97% is greater than the proposed species cutoff of 95% (Thompson et al., 2013). The dendrogram of a distance matrix representing the product of ANI and percentage of the genome alignment created a clustermap that is similar to the rooted Phylogenetic tree with BP-1(T) and BP-2 separating the *Escherichia, Shigella, Citrobacter* and *Salmonella* clade from the *Klebsiella, Kluyvera, Enterobacter, Siccibacter* and *Kosakonia* branch (Figure 4). While the ordering of the two *Citrobacter* clades, *Salmonella* and *Escherichia/Shigella* is similar between the ANI dendrogram and the core genome phylogenetic tree, in the ANI dendrogram *Kosakonia* and not *Kluyvera*/*Klebsiella* is the closest genera to BP-1(T)/BP-2.

### Phenotypic antibiotic susceptibility and antibiotic resistance gene analysis

Both strains BP-1(T) and BP-2 had phenotypic resistance to all cephalosporins tested except cefepime and cefotetan (Table 5). This susceptibility profile suggests carriage of an extended-spectrum β-lactamase (ESBL). Both isolates were resistant to ampicillin and aztreonam but susceptible to carbapenems (meropenem and imipenem) and β-lactam/β-lactamase inhibitor combinations (pipercillin-tazobactam, ceftazidime-avibactam and ampicillin-sulbactam; Table 5). The isolates were resistant to gentamicin but susceptible to amikacin (Table 5). Each isolate was additionally resistant to trimethoprim-sulfamethoxazole and susceptible to doxycycline, minocycline and tigecycline (Table 5). BP-1(T) was susceptible to fosfomycin but BP-2 had intermediate resistance (Table 5). Both isolates shared the same antibiotic resistance genes. ResFinder identified *bla*_SHV−12_, *bla*_TEM−1b_, *qnrB2, aac(6*′*)IB-cr, strB, aac(6*′*)-IIC, aacA4, aph(3*′*)-Ia, strA, sul1, sul2, dfrA18, catA2* and *ere*(A) (Table 5). Resfams identified the above genes except for *sul1, sul2* and *dfrA18*, but found an additional Class A β-lactamase (Table 5). In BP-1(T) the class A β-lactamase was on contig 4, believed to be chromosomal as the highest blastn match was the complete genome of *C. amalonaticus* FDAARGOS and had 96% identity with 68% query coverage to a HER family β-lactamase (WP_023281079.1). Preceding it was a tetR response regulator with similarity to a protein from *Klebsiella* sp. RIT-PI-d (WP_049839935.1).

**Table 5:** Phenotypic antibiotic susceptibility and putative resistance determinants.

	**BP-1(T)**		**BP-2**	
**Antibiotic**	**Phenotypic resistance**	**Putative resistant determinant**	**Phenotypic resistance**	**Putative resistant determinant**
Aztreonam	R	*bla*_SHV−12_, *bla*_TEM−1b_, Class A	R	*bla*_SHV−12_, *bla*_TEM−1b_, Class A
Ampicillin	R		R	
Cefazolin	R		R	
Cefotetan	S		S	
Ceftriaxone	R		R	
Ceftazidime	R		R	
Cefepime	S		S	
Meropenem	S		S	
Imipenem	S		S	
Pipercillin-Tazobactam	S		S	
Ceftazidime-Avibactam	S		S	
Ampicillin-Sulbactam	S		S	
Ciprofloxacin	S	QnrB2, aac(6′)IB-cr	S	QnrB2, aac(6′)IB-cr
Levofloxacin	S	QnrB2	S	QnrB2
Gentamicin	R	strB, aac(6′)-IIC, aacA4, aph(3′)-Ia, strA, aac(6')Ib-cr	R	strB, aac(6′)-IIC, aacA4, aph(3′)-Ia, strA, aac(6′)Ib-cr
Amikacin	S		S	
Trimethoprim-sulfamethoxazole	R	sul1, sul2, dfrA18	R	sul1, sul2, dfrA18
Fosfomycin	S		I	
Doxycycline	S		S	
Minocycline	S		S	
Tigecycline	S		S	
Nitrofurantoin	S		S	
Not tested	N/A	catA2, ere(A)	N/A	catA2, ere(A)

**Figure 5:**
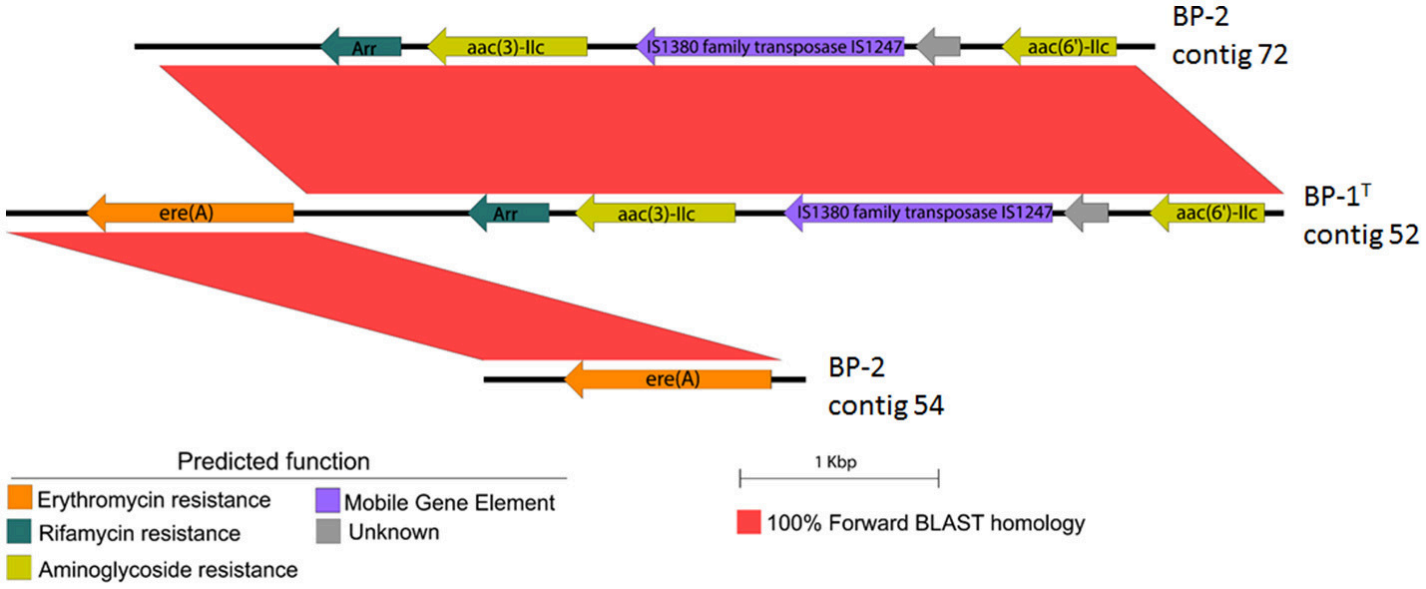
EasyFig construction showing BLAST-based sequence similarity between genetic structure of transposase and putative ARGs on the same contig in BP-1(T) and two different contigs in BP-2. Scale bar represents sequence length.

To gain further insight into the potential for antibiotic resistance gene dissemination from strains BP-1(T) and BP-2, we analyzed the draft genomes of BP-1(T) and BP-2 for contigs that had ARGs co-localized with mobile gene elements. An IS1380 family transposase was found co-localized in both genomes with an erythromycin resistance gene ere(A), a putative rifampin resistance gene that was unidentified by ResFinder or Resfams, a putative aminoglycoside N-acetyltransferase unidentified by Resfinder or Resfams, a hypothetical protein and aac(6′)-IIC (Figure 5).

## Discussion

The purpose of our investigation was to determine the taxonomic identity of strains BP-1(T) and BP-2, two isolates recovered from selective culturing of swabs of two Pakistani ICU room surfaces which could not be identified using MALDI-ToF mass spectroscopy. The colony morphology of strains BP-1(T) and BP-2 on blood agar is indistinguishable from non-swarming Enterobacteriaceae, but the results of growth on MacConkey plates and ChromID ESBL agar show phenotypic similarities to lactose fermenting and extended spectrum β-lactamase producing species such as *E. coli* and *K. pneumoniae* (Grohs et al., 2013). Ultrastructural analysis showed morphological and size similarity to other Enterobacteriaceae species (Feng et al., 2014). Consistent with the observed lack of a flagellum, strains BP-1(T) and BP-2 were non-motile like *Yersinia, Klebsiella* and *Shigella* genera of Enterobacteriaceae (Kramer et al., 2006).

Differential growth assays and API results were not able to assign strains BP-1(T) and BP-2 to an existing genus with high confidence. API testing determined that both isolates share a unique suite of phenotypes that distinguish BP-1(T) and BP-2 from other Enterobacteriaceae genera. The closest identity to strains BP-1(T) and BP-2 based on biochemical utilization and API algorithm predictions were *Kluyvera* species (41.2%), *E. cloacae* (30.7%) and *Citrobacter koseri*/*farmeri* (8.4%). Strains BP-1(T) and BP-2 differed from these most closely related taxa by API in arginine dihydrolase, indole production, acetoin production, sorbitol fermentation and motility.

To further probe the appropriate classification of BP-1(T) and BP-2, we performed Illumina whole genome sequencing on both isolates. As the BP-1(T) 16S rRNA gene sequence is 98.7% identical to the *C. farmeri* CDC 2991-81 rRNA gene, we speculated that these isolates may represent a novel species. To gain greater taxonomic resolution beyond the limitations of using just the 16S rRNA sequence, we identified and aligned all shared protein coding sequences between strains BP-1(T), BP-2 and type strains from *Enterobacter, Klebsiella, Kluyvera, Citrobacter, Kosakonia, Escherichia, Shigella, Salmonella*, and *Siccibacter*. Although the BP-1 ^T^ 16S rRNA sequence had the greatest similarity to several *Citrobacter* species, the unrooted NeighborNet tree and rooted maximum likelihood tree indicate BP-1(T) and BP-2 form a cluster separate from known Enterobacteriaceae species. A rooted phylogenetic tree placed the taxonomic position of BP-1(T) and BP-2 in between two large clades; one clade contains *Escherichia, Shigella, Salmonella* and *Citrobacter* and the other contains *Klebsiella, Kluyvera, Enterobacter, Siccibacter* and *Kosakonia*. ANI is the accepted *in silico* version of a DNA-DNA hybridization assay, the gold standard for demarcation of new bacterial species (Richter and Rossello-Mora, 2009). The 99.97% ANI between strains BP-1(T) and BP-2 indicate that they are the same species and possibly clonal isolates. A dendrogram of the ANI output reflected a tree topology like the rooted core genome tree but had *Kosakonia* and not *Kluyvera*/*Klebsiella* as the closest genera to BP-1(T) and BP-2.

Both strains BP-1(T) and BP-2 are multidrug resistant Enterobacteriaceae which harbor extended spectrum β-lactamases and are therefore classified as a serious antimicrobial threat by the CDC (CDC, 2013; Iredell et al., 2016). The Class A β-lactamase identified by Resfams had high identity to *bla*_HER_ family genes, which were previously described in *Atlantibacter hermanii* but only conferred resistance to penicillins (Beauchef-Havard et al., 2003). As this gene was proximal to a tetR response regulator, it is possible that it may be inducible, like the ampR/ampC system widespread in *Enterobacter* and *Citrobacter* (Vadlamani et al., 2015). However further work comparing the expression levels under basal or induced conditions would be needed to ascertain this. TEM-1 β-lactamase genes are widespread in *E. coli* and *K. pneumoniae* and can confer resistance to penicillins and first/second generation cephalosporins, but not 3rd generation drugs like ceftazidime (Cantu et al., 1997). *bla*_TEM−1b_, the variant found in strains BP-1(T) and BP-2 is not considered an ESBL (Udomsantisuk et al., 2011). Therefore, a combination of blaTEM-1 and *bla*_SHV−12_ are therefore likely the factor most critical for cephalosporin resistance (Teshager et al., 2000; Newire et al., 2013). However, efflux and porin activity may augment β-lactamase production to achieve clinical resistance, as seen in other Enterobacteriaceae species (Wozniak et al., 2012; Taherpour and Hashemi, 2013). Several predicted antibiotic resistance genes in these genomes were co-localized with IS1247, an IS1380 family transposase. A previously discovered IS1380 family member, ISEcp1 can transfer *bla*_CTX−M_ via transposition (Poirel et al., 2005; Toleman et al., 2006).

One limitation of our study is that we have only procured two strains and given the temporal similarity they may be clonally related. Although not ideal from a taxonomic perspective, the Enterobacteriaceae species *Klebsiella michiganensis, Enterobacter soli, Enterobacter muelleri* and genera *Chania* and *Nissabacter* were all established from single strain investigations (Manter et al., 2011; Saha et al., 2013; Kampfer et al., 2015; Ee et al., 2016; Mlaga et al., 2017). This study further demonstrates the utility of whole genome sequencing for pathogen identification in a clinical setting. Similarity, another report found that Vitek2 identified a blood culture derived isolate as *E. cloacae* but Illumina whole genome sequencing unequivocally determined it to be *Kosakonia radicincitans* (Bhatti et al., 2017). Further comprehensive work is therefore warranted to investigate clinical outcomes that result from these species misidentification.

Further work using long-read assembly such as PacBio or Oxford Nanopore is warranted to construct a high-quality complete genome. These additional efforts may also unambiguously identify plasmid components that replicate independently of the chromosome but could be collapsed into the same fasta file using our draft genome approach. This may additionally inform the presence of specific ARGs as chromosomal or plasmid-borne. Unfortunately, these techniques were not available to us during the course of this investigation.

### Description of *superficieibacter* gen. nov.

*Superficieibacter* (Su.per.fi.ci.e.i.bac′ter. L. fem. n. *superficies* surface; N.L. masc. n. *bacter* a rod: N.L. masc. n. *Superficieibacter* a rod from a surface).

Cells are facultative aerobic Gram-negative bacilli visualized without chains but occasionally in clusters under electron microscopy. Non-motile with no evidence of a flagella. Cells are mesophilic with robust growth at 35°C, scant growth at 25 and 32°C and no growth at 4°C. Cells can grow anaerobically at 35°C. Cells are catalase positive and can produce gas in the TSI and LIA slants. Cells can ferment arabinose, amygdalin, melibiose, sacharose, rhamnose, sorbitol, lactose, glucose and mannitol. Strains can be differed from related genera by being negative for arginine dihydrolase, indole production, acetoin production and motility but positive for sorbitol fermentation. Members belong to class Gammaproteobacteria, order Enterobacteriales and family Enterobacteriaceae. The type species of the genus is *Superficieibacter electus*.

### Description of *superficieibacter electus* sp. nov.

*Superficieibacter electus* (e.lec'tus. L. part. adj. *electus* chosen).

Main attributes are applicable from genus. Colonies form: circular, shiny, mucoid, non-haemolytic gray colonies on blood agar plates; small, blue and rigid colonies on HardyCHROM™ ESBL Agar; bright pink colonies on MacConkey agar; Coral colonies on HE agar. Cells are rod shaped and ~2.7 μm in length. 16S rRNA sequence of the strain BP-1(T) had highest percentage identity to *C. farmeri* CDC 2991-81(T) (98.63%) *C. amalonaticus* CECT 863(T) (98.56%), *Citrobacter sedlakii* NBRC 105722(T) (97.74%) and *Citrobacter rodentium* NBRC 105723(T) (97.74%). Strain BP-1(T) and BP-2 form a cluster with each other based on core genome phylogeny with related genera in the family Enterobacteriaceae. GC percentage of strain BP-1(T) and BP-2 is 52.4%. The type strain is BP-1(T) (= ATCC BAA-2937, = NBRC 113412).

## Author contributions

RP and AD wrote the manuscript and performed *in silico* analysis. MW and AS performed culture work, growth assays and API test. DG isolated the bacteria from hospital surfaces. SP extracted genomic DNA and prepared Illumina sequencing libraries. WB performed electron microscopy. JK, SA, C-AB and GD devised the study.

### Conflict of interest statement

The authors declare that the research was conducted in the absence of any commercial or financial relationships that could be construed as a potential conflict of interest.
