# Supplementary figures and images for: Superficieibacter electus gen. nov., sp. nov., an Extended-Spectrum β-Lactamase Possessing Member of the Enterobacteriaceae Family, Isolated From Intensive Care Unit Surfaces
Source: Front Microbiol. 2018 Jul 20;9:1629. doi: 10.3389/fmicb.2018.01629 (PMC6062592; doi:10.3389/fmicb.2018.01629)

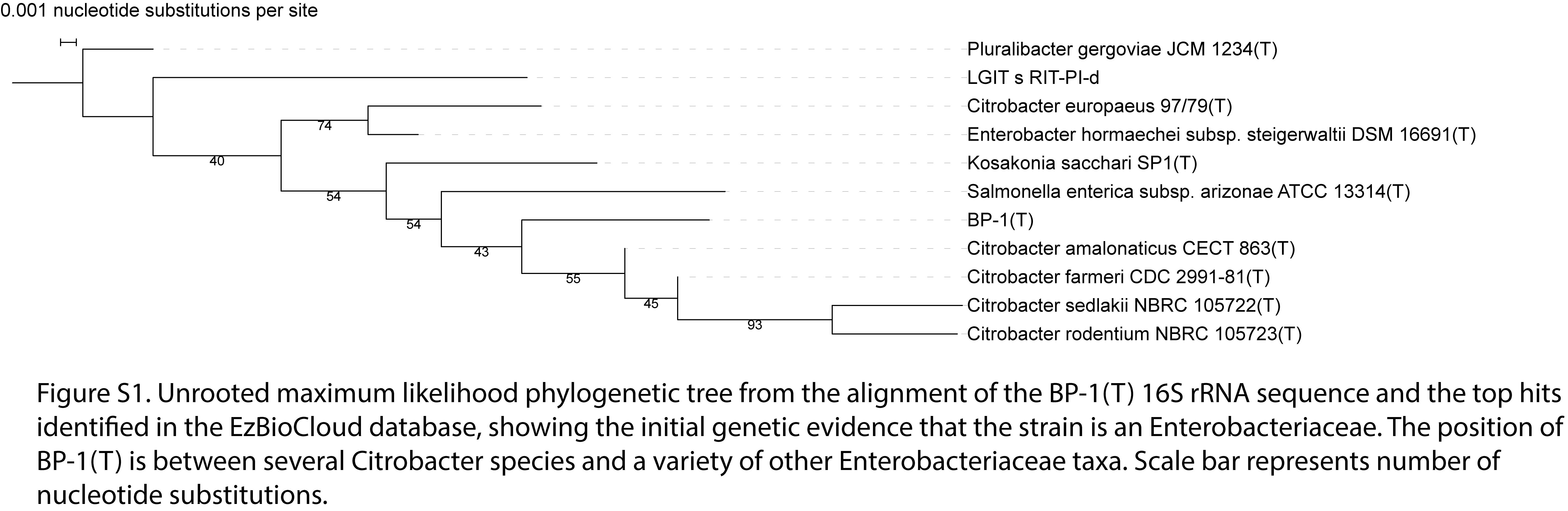

Supplement: Supplementary file 1 [file Image_1.tif]

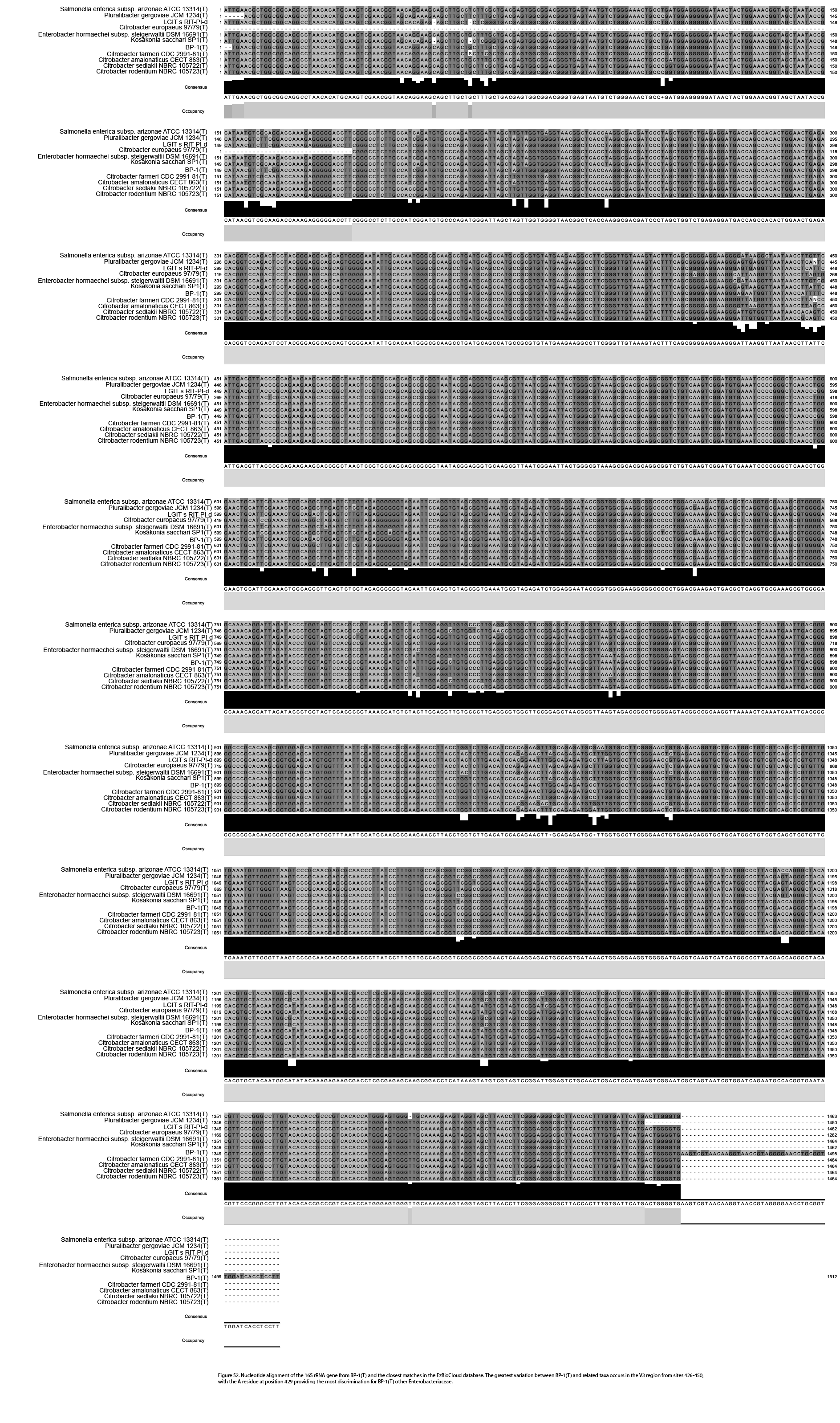

Supplement: Supplementary file 2 [file Image_2.TIFF]
